# Supplementary material for: The History and Prediction of Prebiotics and Postbiotics: A Patent Analysis
Source: Nutrients. 2024 Jan 27;16(3):380. doi: 10.3390/nu16030380 (PMC10857523; doi:10.3390/nu16030380)
Supplement: Supplementary file 1 [file nutrients-16-00380-s001.zip › nutrients-2783928-supplementary.pdf]

Table S1. Information on the functional role of prebiotics

Functionality	Firms	Patent Number	Skill
Amino acid Metabolism	Sigmoid Pharma Limited	US20060018965 A1	Controlled Release Microcapsules
Amino acid Metabolism	Hoelzel, Karl Heinz, 8081 Schoengeising, De	DE4033996 A1	Probiotics Enzymatic Metabolites Capsules
Amino acid Metabolism	Nestlé Nestec Sa	WO2010000776 A1	Temperature-sensitive microorganisms and their use in delivering compounds to specific regions of the body
Amino acid Metabolism	Jiangnan University	CN111700904 A	Use of acylated starch for the targeted release of short-chain fatty acids in the large intestine
Amino acid Metabolism	Guangzhou University	CN104667285 B	Semi-trellised-polydopamine material slow-release carriers
Amino acid Metabolism	Pfizer Pharmaln Corporation	US9737615 B2/ CN101365490 B	Biocompatible hydrophobic core carriers
Amino acid Metabolism	The Regents Of The University Of California	WO2020014106 A1/ CN112384623 A	Protein-based micelles for delivery of hydrophobically active compounds
Amino acid Metabolism	Massachusetts Institute Of Technology; The Brigham And Women's Hospital, Inc.	US10716751 B2/CN106573999 A	Modular design regulates residence time structure
Amino acid Metabolism	Japan Science And Technology Corporation	WO03000285 A1	Tissue-specific transporter inhibitors containing supramolecularly structured polyrotaxanes
Amino acid Metabolism	Intrexon Arctubio Dix Ltd. Intrexon Actobiotics N.v.	KR20190056387 A/ CN109983028A	Microorganisms with enhanced cell adhesion properties regulate microbial gastrointestinal retention time
Amino acid Metabolism	Massachusetts Institute Of Technology; The Brigham And Women's Hospital, Inc.	CN106573015 A	Hydroxyl-containing polymer enteric elastomer composites utilising hydrogen bonding
Amino acid Metabolism	Cell Biotech Co., Ltd.	KR20190135343 A	Fusion proteins that bind to targeted peptides and active ingredients

Table S1. Information on the functional role of prebiotics

Functionality	Firms	Patent Number	Skill
Amino acid Metabolism	State Of Oregon Acting By And Through The State Board Of Higher Education On Behalf Of Oregon State University	US20040219186 A1/CN1543337 A	Gastric retention device comprising a polysaccharide mixture forming a soluble polymer gel
Amino acid Metabolism	Yeda Research And Development Co., Ltd.	US20100135974 A1	Redirected genetically engineered t-regulatory cells
Amino acid Metabolism	Rhone-poulenc Nutrition Animale	US4181710 A/US4181708 A	Coating for resistance to the rumen environment
Amino acid Metabolism	Adare Pharmaceuticals, Inc.	US20070196491 A1	Controlled release dosage forms of target-delivered drugs with multiple coatings
Amino acid Metabolism	Hisamitsu Pharmaceutical Co., Inc	EP1607087 A1	Double-coated formulations consisting of anionic and cationic polymers
Amino acid Metabolism	Lek, Tovarna Farmacevtskih In Kemicnih Izdelkov, D. D.	SK282000 B6	Three-phase controlled release dosage form and its preparation method
Amino acid Metabolism	Dexcel Pharma Technologies Ltd.	PL203733 B1/CN100548279 C	Delayed release drug systems
Amino acid Metabolism	Flamme Technologies	JP5687185 B2	Dosage forms with release controlled by both time and pH
Amino acid Metabolism	Nestlé Nestec Sa	CN104206946 A	Dietary fibre, oligofructose and other formulations
Amino acid Metabolism	Huguet Helene-celine; Andremont Antoine; Tsapis Nicolas; Fattal Elias	US20120107367 A1	Programmed delivery system to the lower part of the small intestine
Amino acid Metabolism	PLx pharmaceutical company / Plx Opco Inc.（美国德克萨斯州(TX)）	US9730884 B2/ CN103957888 B	Bioactive or pH-dependent targeted delivery carriers containing free fatty acids (FFA)
Amino acid Metabolism	Caledo Biotechnology Co. kaleido biosciences inc	JP2018502926 A/ CN107427528 A	Glycan therapeutic agents, microbiome modifiers

Table S1. Information on the functional role of prebiotics

Functionality	Firms	Patent Number	Skill
Amino acid Metabolism	Aptalis Pharmatech, Inc.(Teva pharmaceutical industries ltd.)	US20110135724 A1	Controlled release orally disintegrating tablets
Amino acid Metabolism	Adare Pharmaceuticals, Inc.	US20070190145 A1	Controlled release dosage forms of target-delivered drugs with multiple coatings
Amino acid Metabolism	The Royal Institution For The Advancement Of Learning (mcgill University)	US5147641 A	Microencapsulated dosage form containing enzymes targeting intestinal amino acids
Amino acid Metabolism	Golf Acquiror LLC	US20120064168 A1	Gastric retention dosage form of gabepentin with semi-permeable membrane
Amino acid Metabolism	ndustry Foundation Of Chonnam National University	US10034903 B2	Microbots for bacteria, microbeads
Polyphenol	Zhejiang Wecome Medicine Industry Co., Ltd.	CN110251478 A	High temperature resistant enteric coating
Polyphenol	希格默伊德药业有限公司	JP2010523553 A	Contains cyclosporine mini capsules
Polyphenol	Anji Pharma (us) LLC	US20120177730 A1	chemosensory receptor ligand
Polyphenol	Toyo Shinyaku Co Ltd	JP2015059094 A	Effective promotion of catechin binding to cells by increasing the expression level of the gene encoding catechin receptor protein 67LR
Polyphenol	Zhong Shuguang	CN101987081 A	controlled release formulation
Polyphenol	Everest Pharm. Industrial Co., Ltd.	TWM601104 U	Crystalline spherical multilayer structure
Polyphenol	Qingdao Agricultural Unive	CN105852060 B	Starch-polyphenol composite nanoparticles
Polyphenol	Anji Pharma (us) LLC	US20150065578 A1	Delayed release (DR) formulations
Polyphenol	Hao Zhang	US20030166508 A1	Absorbent promoter, stabiliser, and specific capsule shells
Polyphenol	Johnson Chemical Pharmaceutical Factory Co., Ltd.	CN110339365 A	drug formulation

Table S1. Information on the functional role of prebiotics

Functionality	Firms	Patent Number	Skill
Purine Metabolism	Bristol-myers Squibb Co	NO330554 B1	Enteroprotective coating containing methacrylic acid
Purine Metabolism	Sigmoid Pharma Limited	EP2409691 A1	Microcapsules for delivery of cyclosporin a to the colon
Purine Metabolism	Conaris Research Institute Ag; Christian-albrechts-universit?t Zu Kiel	WO2017182347 A1	Bi-layer microencapsulation system for pH targeting
Purine Metabolism	Ip Science Limited （Cambridge, England）	UA115121 C2/ CN107028936A	Increased bioavailability of cargo molecules through carotenoid particle loading
Purine Metabolism	Alza Corporation	JP2006507309 A	Emulsified nanosuspension formulations
Purine Metabolism	Bell Juk Kuang	CN101987081 B	Controlled release dosage forms based on polymer coating film positioning
Purine Metabolism	Deloitte Pharmaceuticals Ltd. Tillotts Pharma Ag	CU24302 B1/ CN104271113 A	Bilayer pH III polymeric material coated formulations
Purine Metabolism	CG Association/Sparks USA (NV, Washoe County)	MXPA01010918 A	Compositions for delivering hexadecyl myristate to the gut
Purine Metabolism	GHOSH PRASANTA KUMAR , GUPTA VIPIN BIHARI , RATHORE MAHENDRA SINGH , GONDALIYA BHAVIK	IN2009MU02548 A	Biodegradable polymer formulations for drug delivery to the colon
Purine Metabolism	Berkemeyer, USA Birkmayer U.s.a.	AU674583 B2	NADH pills with acid-stable protective coating
Purine Metabolism	Kibo Biotech	KR20040029995 A/ CN100337549 C	formulas
Purine Metabolism	Conaris Research Institute Ag	KR20160088436 A	Pharmaceutical formulations containing 5-aminosalicylic acid and nicotinamide
Purine Metabolism	Schering-Plough Health Care Products, Inc.	TW200815050 A	Phenylephrine intestinal target delivery formulations

Table S1. Information on the functional role of prebiotics

Functionality	Firms	Patent Number	Skill
Other Metabolism	Hunan Jingtian Technology Industry Co.	CN106173265 A	Enteric coated formic acid
Other Metabolism	Hunan Jingtian Technology Industry Co.	CN106107055 A	Enteric coated butyric acid preparation
Other Metabolism	Aizawa Seiki Co.	CN1972698 B	formulas
Other Prebiotics	Miller Guy W.	US20040091537 A1	Fructose-based coating formulations for indigestible carbohydrates
Other Prebiotics	British Sugar Plc	GB2367002 A	Coating formulations containing water-insoluble film-forming polymer matrices and dietary fibres
Other Prebiotics	Alpiflor S.r.l.	EP2289505 B1	Probiotic carrier formulation with probiotics and butyric acid compounds
Other Prebiotics	Funda, Elger;Krainz, Odile;Steinert, Robert	JP2021501565 A	Multi-particle delivery system with inner and outer coating
Other Prebiotics	University Of Southern California	US10631564 B2	Multi-layer particulate dosage form containing alginate, starch acetate and protein
Other Prebiotics	Mcgill University	WO2007140613 A1	Microencapsulated formulations of milk carriers containing bacteria and fermentation
Other Prebiotics	Anhui Zhengdayuan Feed Co., Ltd.	CN103005157 B	Microencapsulated formula with probiotics and prebiotics
Other Prebiotics	Ausa Pharmed Ltd.; Folate (shenzhen) Precision Nutrition Food Group Co., Ltd.; Shenzhen Changqing Medical Science Research Institute	CN112168802 A	Enteric capsules based on tamarind gum
Other Prebiotics	Commonwealth Scientific & Industrial Research Organisation	JP6033998 B2	Microencapsulated dosage forms containing film-forming proteins and carbohydrates

Table S1. Information on the functional role of prebiotics

Functionality	Firms	Patent Number	Skill
Other Prebiotics	Inner Mongolia Agricultural University	CN105105144 B	Preparation of probiotic microcapsules containing rennet enzyme
Other Prebiotics	Jinling Institute Of Technology	CN104435283 B	Preparation of microcapsules containing sodium alginate with protein wall materials
Other Prebiotics	Universidade Federal De Pelotas	BR102018068723 A2	Microcapsules containing xanthan gum
Other Prebiotics	University Of Saskatchewan	WO2015019307 A1	Microcapsules containing biopolymers, plant proteins
Other Prebiotics	Shaoxing Tongchuang Biotechnology Co., Ltd.	CN109700781 A	Stable microspheres formed by liquid spraying and cross-linking with divalent calcium metal ions
Other Prebiotics	Fundacion Leia, C.d.t.	ES2350436 B1	microencapsulated symbiotic material
Other Prebiotics	Chengdu New Keli Chemical Technology Co., Ltd.	CN108936680 A	Web Microspherical Gluten Probiotic Capsules
Other Prebiotics	Beland, Genevieve; Barbeau, Julie; Fliss, Ismail	CA2657291 A1	Beneficial biomass carriers based on maple extracts
Other Prebiotics	Biomatrix, Inc	WO02085415 A1	Fructose-based icing formulations
Other Prebiotics	Ewos Innovation As	NO20150715 A1	Delivery system containing ethylenediammonium alginate
Other Prebiotics	Mogon Pharmaceuticals Sagl	RU2692473 C1	Slow release compositions with hydroxypropyl methyl cellulose matrix embedded in curcumin
Other Prebiotics	Northeast Agricultural University	CN104543611 A	Microencapsulated Dosage Forms Containing Oligosaccharides Melade Products
Other Prebiotics	Zhejiang University Of Technology	CN110547468 A	Probiotic microcapsules containing edible mushroom oligosaccharides
Other Prebiotics	Cj Cheiljedang Corporation	KR20170072825 A	Alginate-calcium beads coated with lactic acid bacteria
Other Prebiotics	The University Of Hull	CN101917979 B	Protective preparations for the outer shell of naturally occurring spores
Other Prebiotics	Beijing Technology And Business University	CN110251474 A	Starch-based colon-targeted bi-layer probiotic tablets

Table S1. Information on the functional role of prebiotics

Functionality	Firms	Patent Number	Skill
Other Prebiotics	Dsm Ip Assets B.v.	WO2020043749 A1	Enzyme Delivery Systems
Other Prebiotics	Socit Des Produits Nestl S.a.	US7101565 B2	Mucopolysaccharide prebiotic compositions formulation
Other Prebiotics	Metamodix, Inc.	US10159699 B2	Systems affecting the intestinal microflora
Other Prebiotics	Takaki Yasumura;Hidesaku Yamamoto;Natsuki Morishita	JP2005526861 A	Methods for controlling the rate of methane manipulation for gastrointestinal transport
Other Prebiotics	Glycologic Limited	AU2006340298 A1	Starch-based gastric floatation compositions
Other Prebiotics	Kabadi, Mohan; Schentag, Jerome, J.	WO2014152338 A1	A microencapsulated live probiotic-targeted delivery system with a two-stage release pattern
Other Prebiotics	Only Co., Ltd., Shanghai Jiantong Univ.	CN109464425 A	Triple probiotic encapsulated pellets
Other Prebiotics	Purdue Research Foundation	US20140179629 A1	Starch-containing cross-linked polymer particles
Other Prebiotics	Shandong Tanke Biological Technology Co., Ltd.	CN109674061 A	Stabilised double-layer microencapsulation with sodium alginate and poly-L-arginine
Other Prebiotics	Therabiome LLC; Schentag Jerome J.	IN2015DN07705 A	Targeted gastrointestinal delivery
Other Prebiotics	Yoshiyuki Inaba; Toshifumi Ogun; Akihiko Eguchi; Kazuhiko Naito	JP2016517425 A	Capsule Encapsulated Targeted Delivery System
Other Prebiotics	Sato, Tsuyoshi; Sakata, Keiji; Yamazaki, Hiroshi; Tanaka, Mitsuo; Yano, Masaki	JP2011524890 A	Multi-layer, multi-site drug delivery dosage forms
Other Prebiotics	Centro Nacional De Tecnolog?a Y Seguridad Alimentaria, Laboratorio Del Ebro; Universidad De Navarra	BR112015000041 A2	Micronised formula with casein and chitosan and prebiotics
Other Prebiotics	Evonik Operations Gmbh	EP3646857 A1	Colonic Delivery Capsule Formulation

Table S1. Information on the functional role of prebiotics

Functionality	Firms	Patent Number	Skill
Other Prebiotics	Iker, Brandon C;Hillebrand, Greg;	JP2020504159 A	Compositions containing resistant starch and polysaccharides
Other Prebiotics	Ocean University Of China	CN111820419 A	Prebiotic formulation based on galactomannan and fucoidan complexes
Other Prebiotics	Ohio State Innovation Foundation	US20180000878 A1	Microspheres containing biofilm-producing probiotics, prebiotics or prebiotic membranes
Other Prebiotics	Saito Ryozo	JP2016029065 A	Formulation with probiotic generators
Other Prebiotics	Zhejiang Huakang Pharmaceutical Co., Ltd.	CN109497555 A	Microcapsules containing acacia sugar, pH-triggered materials and colony-triggered materials
Pentacyclic Triterpenoids	Benzon Pharma A/s Te Hvidovre, Denemarken	EP0080341 A2	Corrosive coating resistant to gastric fluids
Pentacyclic Triterpenoids	Perry Bruce	US20190008780 A1	Dual-action delivery of hepatic supplements to the small intestine by exocapsules and endocapsules
Fatty acid Metabolism	Shanghai Institute of Pharmaceutical Sciences, Chinese Academy of Sciences	CN108653234 B	Dual enteric granule formulations containing protease preparations
Fatty acid Metabolism	Sigmoid Pharmaceuticals Ltd. （Sigmoid Pharma Limited）	CN107106644 A	Polymer-coated formulations containing hydrogels
Fatty acid Metabolism	Sigmoid Pharmaceuticals Ltd. （Sigmoid Pharma Limited）	CA2683415 C	Microcapsules of mixed shell/core suspension
Fatty acid Metabolism	Hewili Glaxo Group Limited	WO2007012478 A2	Enteric microcapsules
Fatty acid Metabolism	Hewili Glaxo Group Limited	WO2005070401 A1	Enteric soluble microcapsules containing lipid-holding carriers
Fatty acid Metabolism	Biolek Sp. Z O.o.	EP2597972 A1	Formulated with probiotics and short-chain fatty acids

Table S1. Information on the functional role of prebiotics

Functionality	Firms	Patent Number	Skill
Fatty acid Metabolism	Farmacia, Sweden Pharmacia & Upjohn Ab	JPH08507515 A	Delivery system for isometric shaped colloidal solid lipid particles
Fatty acid Metabolism	DSM Intellectual Property Assets Ltd.（Dsm Ip Assets B.v.）	JP2021501565 A	Double Coated Nutrient Delivery Systems
Fatty acid Metabolism	DSM Intellectual Property Assets Ltd.（Dsm Ip Assets B.v.）	WO2020043749 A1	Two-tier delivery system
Fatty acid Metabolism	Hubei Huayang Technology Development Co.	CN106490345 A	Preparation of acidifying agents for lipid-containing and short-chain fatty acid-containing envelope layers
Fatty acid Metabolism	Nestlé Products Technical Assistance Ltd.	CN102595934 A	formulas
Fatty acid Metabolism	Nestlé Products Technical Assistance Ltd.	RU2008116057 A	Dietary fibre preparations and methods of administration
Fatty acid Metabolism	The University Of New South Wales；Corn Products Development, Inc.	US6348452 B1	Formulated with resistant starch and probiotics
Fatty acid Metabolism	Vedanta Biosciences, Inc.	US9764019 B2	Formulated with small molecules and bacterial antigens
Fatty acid Metabolism	Ocean University of China	CN111820419 A	Formulation for the targeted regulation of Eckermannia intestinalis and short-chain fatty acid-producing bacteria
Fatty acid Metabolism	Finch Therapeutics Holdings LLC	WO2021016083 A1	Compositions for the protection of the Gl microbiome of the gastrointestinal tract
